# Examinations in Tropical Medicine

**Published:** 1904-11-26

**Authors:** 


### Examinations in Tropical Medicine.

It is interesting to watch the steady growth of anything whether animate or inanimate, concrete or abstract. In our own times public health, bacteriology, and colonial medicine have grown from the smallest beginnings to a very satisfactory and substantial size. In each case the science has rapidly become specialised, and has given employment to a number of medical men who would otherwise have continued in the routine of ordinary practice. The specialism has been all for good because it has attracted men of high calibre and great enthusiasm who have quickly started flourishing schools of pupils. Colonial medicine has been even more fortunate because it has succeeded in obtaining the patronage of an active and enlightened Colonial Secretary who, with rare foresight, has fostered its growth in every possible manner. During the last year much consideration has been devoted to the question of instituting an examination and diploma in tropical medicine and hygiene. The University of Cambridge has already established such an examination and diploma, and at the annual meeting of Fellows and Members of the Royal College of Surgeons of England the president explained what steps had been taken to establish a similar examination in London. The attention of the Royal College of Physicians and Surgeons was first called to the matter by a communication from the Colonial Secretary. The question was referred to the joint committee of management of the two colleges, which, while expressing opinions in many respects adverse to the proposal, nevertheless recommended that "after a sufficient period of observation of such tropical diseases, candidates should be admitted to an examination held by the Royal Colleges, and on passing it should receive a diploma in testimony of this addition to their professional experience." This report was not adopted by the Royal College of Physicians and the Council of the Royal College of Surgeons therefore appointed a committee of their own who reported in due course that it was desirable to hold such an examination and grant a special diploma in tropical medicine as had already been done in the case of public health. A conference between representatives of the two colleges took place on July 18th to discuss this resolution and the following recommendations were agreed to and afterwards adopted by the two colleges:—(1) That with the permission of the authorities of the School of Tropical Medicine visitors be appointed by the Royal Colleges of Physicians and Surgeons to attend the examinations of that school and to report upon the scope of the course of study and of examination, and that a request be addressed to the naval, military, and colonial authorities to allow visitors appointed by the two Royal colleges to attend their examinations in tropical medicine for a similar purpose. (2) That at the end of a year a report be addressed by these visitors to the Royal Colleges on the whole subject of tropical medicine. (3) That, in the event of the Royal Colleges adopting these recommendations a communication be sent to the Colonial Secretary informing him of these proceedings.

We venture to hope that there will be a practical issue to the movement thus set on foot, and that, before many years have passed by, those who elect to devote their energies to the study of tropical medicine will be enabled to obtain a diploma representing the knowledge they have acquired in this branch of medicine. Although of modern growth, the study of tropical diseases is perhaps the most important branch of modern medicine. Already the means of preventing some of the most fatal ailments hitherto prevalent in warm climates have been discovered; and every scheme which has for its object the encouragement and equitable treatment of the workers in this field merits the warmest commendation.

